# Genome-Wide Investigation Using sRNA-Seq, Degradome-Seq and Transcriptome-Seq Reveals Regulatory Networks of microRNAs and Their Target Genes in Soybean during *Soybean mosaic virus* Infection

**DOI:** 10.1371/journal.pone.0150582

**Published:** 2016-03-10

**Authors:** Hui Chen, Andrej Adam Arsovski, Kangfu Yu, Aiming Wang

**Affiliations:** 1 Agriculture and Agri-Food Canada, 1391 Sandford ST. London, Ontario, N5T 4T3, Canada; 2 Dept of Biology, The University of Western Ontario, 1151 Richmond ST N. London, Ontario, N6A 5B7, Canada; 3 Greenhouse and Processing Crops Research Centre, Agriculture and Agri-Food Canada, 2585 County Rd. 20, Harrow, Ontario, N0R 1G0, Canada; Kunming University of Science and Technology, CHINA

## Abstract

MicroRNAs (miRNAs) play key roles in a variety of cellular processes through regulation of their target gene expression. Accumulated experimental evidence has demonstrated that infections by viruses are associated with the altered expression profile of miRNAs and their mRNA targets in the host. However, the regulatory network of miRNA-mRNA interactions during viral infection remains largely unknown. In this study, we performed small RNA (sRNA)-seq, degradome-seq and as well as a genome-wide transcriptome analysis to profile the global gene and miRNA expression in soybean following infections by three different *Soybean mosaic virus* (SMV) isolates, L (G2 strain), LRB (G2 strain) and G7 (G7 strain). sRNA-seq analyses revealed a total of 253 soybean miRNAs with a two-fold or greater change in abundance compared with the mock-inoculated control. 125 transcripts were identified as the potential cleavage targets of 105 miRNAs and validated by degradome-seq analyses. Genome-wide transcriptome analysis showed that total 2679 genes are differentially expressed in response to SMV infection including 71 genes predicted as involved in defense response. Finally, complex miRNA-mRNA regulatory networks were derived using the RNAseq, small RNAseq and degradome data. This work represents a comprehensive, global approach to examining virus-host interactions. Genes responsive to SMV infection are identified as are their potential miRNA regulators. Additionally, regulatory changes of the miRNAs themselves are described and the regulatory relationships were supported with degradome data. Taken together these data provide new insights into molecular SMV-soybean interactions and offer candidate miRNAs and their targets for further elucidation of the SMV infection process.

## Introduction

Plant pathogens are a major constraint to agriculture and threaten crop yield and global food security [1,2]. Among diverse plant pathogens, viruses are obligate intracellular parasites that depend on the host cell to provide the basic machinery in order to complete their life cycle [3]. Utilization of genetic resistance is considered the most effective and environmental method for the sustainable control of plant pathogens including viruses [4]. Understanding the molecular mechanisms of virus-host interaction is therefore paramount to developing the next-generation strategies for antiviral resistance in plants. Towards this goal, we launched a comprehensive investigation on the *Soybean mosaic virus* (SMV)-soybean pathosystem using genomic approaches.

SMV, a member of the genus *Potyvirus* in the *Potyviridae* family, is the most prevalent pathogen that impedes soybean production. The viral genome in this largest family of known plant viruses is a positive-sense, single-stranded RNA molecule, approximately 9,600 nucleotides in length. To date, numerous SMV isolates have been reported. Based on their differential responses on susceptible and resistant soybean cultivars, they were classified into seven distinct strains (G1 to G7) [5]. After extensive screening, three independent dominant resistance genes (*Rsv1*, *Rsv3*, and *Rsv4*) with different SMV strain specificities have been identified [6–10]. *Rsv1*, found in soybean cultivar PI96983, confers resistance to the SMV strains G1 to G6 but not to G7 [11,12]. In an earlier study, we reported a naturally occurring *Rsv4* resistance-breaking isolate (SMV-LRB) and a closely related non-resistance-breaking isolate (SMV-L) from Canada [13]. SMV isolates L and LRB belong to the G2 pathotype. Previously, we also explored global gene expression changes of soybean in response to SMV-L infection using microarray [14]. We found a number of genes involved in defence were downregulated or not affected at the early stages of infection but upregulated at the late stages, indicating that the plant immune responses are suppressed or not activated until late in the infection. We speculated that the delayed defence response may be critical for SMV to establish its systemic infection [14].

MicroRNAs (miRNAs) are 20–24 nucleotides in length, single-stranded non-coding RNAs found in all eukaryotes. miRNAs play critical roles in a variety of biological processes such as maintenance of genome integrity, development and feedback mechanisms as well as various biotic and abiotic stress responses [15–17]. miRNAs are transcribed by RNA polymerase II (pol II) and processed by DICER-LIKE (DCL1) protein in the nucleus from stem-loop structures, then interact with Argonaute (AGO) proteins to efficiently form RNA-induced silencing complexes (RISCs) in the cytoplasm and regulate gene expression by translational inhibition or cleavage of complementary mRNAs [18]. In addition to regulating the expression of endogenous genes, miRNAs are also indispensable during innate immune responses in animals and plants. In human cells, miR-32 effectively restricts the accumulation of the retrovirus primate foamy virus type 1 (PFV-1) [19]. miR-122 is specifically expressed and highly abundant in the human liver, and the sequestration of miR-122 into liver cells results in a marked loss of autonomously replicating hepatitis C viral RNAs [20]. In plants, virus infections are often associated with alterations in endogenous miRNAs levels, resulting in changes in the abundance of its target mRNA. The levels of mature miR164, precursor of miR164a and *CUC1* mRNA (a miR164 target) are elevated in *Arabidopsis* plants infected by *Tobacco mosaic virus* Cg (TMV-Cg) or *Oilseed rape mosaic virus* (ORMV) [21]. A screening of over 53 predicted miRNAs in tomato (*Solanum lycopersicum*) plants revealed that many of them are upregulated after infection by *Tomato leaf curl New Delhi virus* (ToLCNDV) [22]. Conversely, some miRNAs in cotton plants are downregulated in response to infection by *Cotton leafroll dwarf virus* (CLRDV) [23]. *Rice dwarf virus* (RDV) and *Rice stripe virus* (RSV) have distinct impacts on rice small RNA metabolism. RSV infection induces the expression of novel phased miRNAs from several conserved miRNA precursors [24]. Several Infections of different hosts by *Cucumber mosaic virus* (CMV, FNY strain) [25], and *Cymbidum ringspot virus* (CymRSV) suggest a direct link between the host immune response and viral infection [26,27]. More recently, we have shown that the accumulation of miR168 and *AGO1* mRNA is significantly induced in *Rsv1* soybean infected by SMV G7, suggesting that both miRNA and siRNA pathways are involved in pathogenesis of SMV G7 in *Rsv1* soybean likely through disruption of AGO1 homeostasis [28].

The development and subsequent affordability of high-throughput DNA sequencing technologies and bioinformatics tools, has led to the identification of a large number of miRNAs and their targets in plants, animals, and viruses. To date, 28,645 hairpin precursor miRNAs, and 35,828 mature miRNAs from 223 species have been annotated and deposited in miRBase (miRBase 21). In *Glycine max*, 573 miRNA precursors and 639 mature miRNAs have been identified from different developmental stages and tissues as well as various biotic and abiotic stress-treated tissues [29–31]. Three highly abundant microRNA families (miR1507, miR2109, and miR2118) are known to target conserved domains in the defense-related NB-LRR-encoding genes and trigger the production of trans-acting siRNAs (tasiRNAs) [32]. Soybean miR172c has been demonstrated to modulates both rhizobium infection and nodule organogenesis [33], whereas miR393 has been identified to be involved in regulating soybean defense in response to *Phytophthora sojae* infection [34]. Three miRNAs (miR160, miR393 and miR1510) have been shown to be involved in the defense response to SMV infection (SC7 isolate in China) [35]. However, most annotations of soybean miRNAs and their targets are derived through computational prediction without experimental validation. The regulatory network of miRNA-mRNA interactions during viral infections including SMV infection still remains largely unknown.

In this work, we employed sRNA-seq, degradome-seq and transcriptome-seq technologies to study the SMV-soybean pathosystem with the aim of identifying common targets of regulation following infection with three SMV isolates. We conducted an integrated analysis of the resulting high throughout data using both bioinformatics tools including target prediction, GO enrichment analyses and interaction networks and experimental approaches, i.e., stem-loop RT-qPCR, RNA blot, RT-qPCR and RLM-5' RACE. We discovered a group of miRNAs and their mRNA targets that were differentially expressed in response to infections by different SMV isolates/strains and identified common miRNA-mRNA regulatory interactions during SMV infections.

## Results

### Common and Unique miRNAs Are Differential Expressed in Response to Infection by Three Different SMV Isolates

To understand the role of soybean miRNAs during SMV infection, we profiled the expression of miRNAs following infection with different strains of SMV in the susceptible soybean cultivar Williams 82 at 14 days post inoculation (dpi). At this time point, the systemic leaves of infected plants showed typical viral symptoms, consistent with our previous observations [13]. Fifteen soybean plants infected by three different isolates/strains or mock-inoculated plants were pooled for RNA extraction and small RNA cDNA library construction. We sequenced four small RNA cDNA libraries (G2-L, G2-LRB, G7 and mock control) using the Illumina deep sequencing technology. After trimming the adapter sequences and removal of low quality reads, a total of 28,135,511 reads were obtained. These reads were mapped to the soybean genome (GLYMA1, Ensembl) and aligned to the known miRNA genes in soybean (miRBase 21) using the Strand NGS software (Strand Life Sciences, version 2.1) following the small RNA alignment and small RNA analysis pipeline. As shown in S1 Table, about 50% reads could be matched perfectly to the soybean genome. This resulted in more than 1.5 million distinct genome-matching small RNA sequences (sRNA-seq) per library. The sRNA-seq from each library was also mapped to each of the 20 soybean chromosomes (S1 Table).

**Fig 1:**
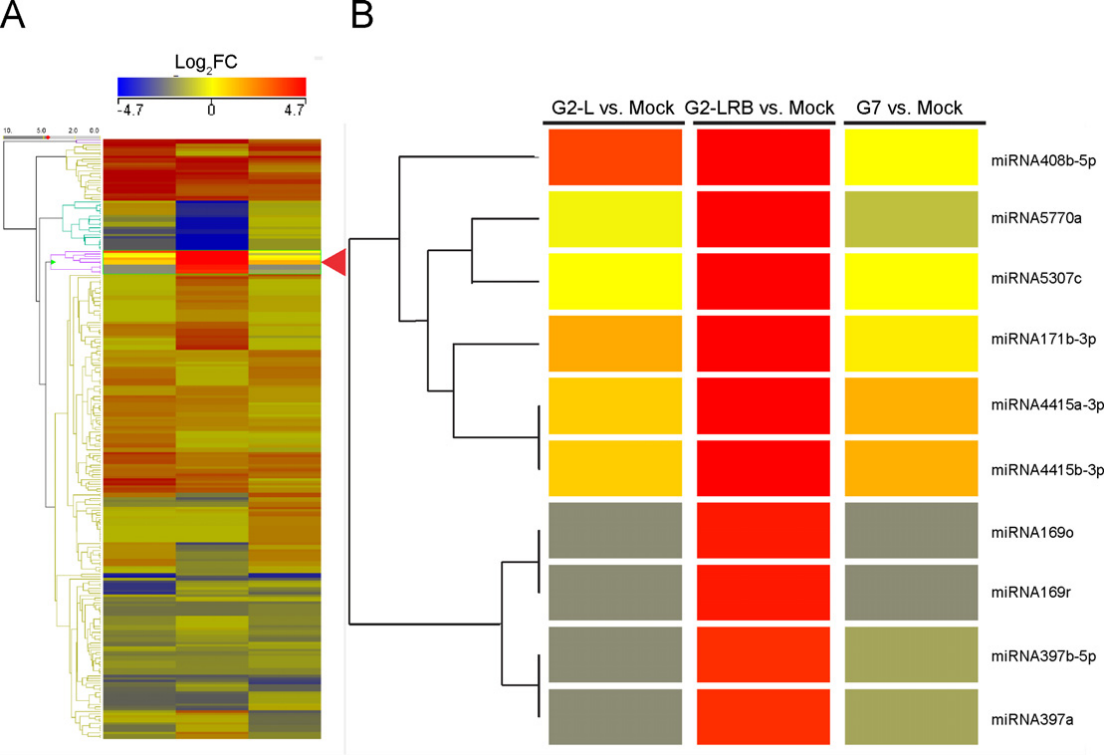
A hierarchical cluster analysis of miRNA expression profiles as a heat map depicting 253 differentially expressed miRNAs (DEMs) after infection by three SMV isolates (G2-L, G2-LRB and G7). **(A)** A summary view of heat map generated using Strand NGS (http://www.strand-ngs.com/). **(B)** A detailed view of an exemplar branch showing DEMs differentially responsive to SMV isolates. Colors indicate the log_2_ fold changes (FC) with a ratio of SMV-infected sample versus mock-inoculated control according to the average of the normalized signal values. All downregulations are indicated with a negative sign and are shown in blue. All upregulations are shown in red colour. Read densities = RPKMs (Read count per kilobase of exon model per million reads); Normalized signal values = log2(RPKMs). Significance was determined by using a fold change threshold of at least 2.

**Fig 2:**
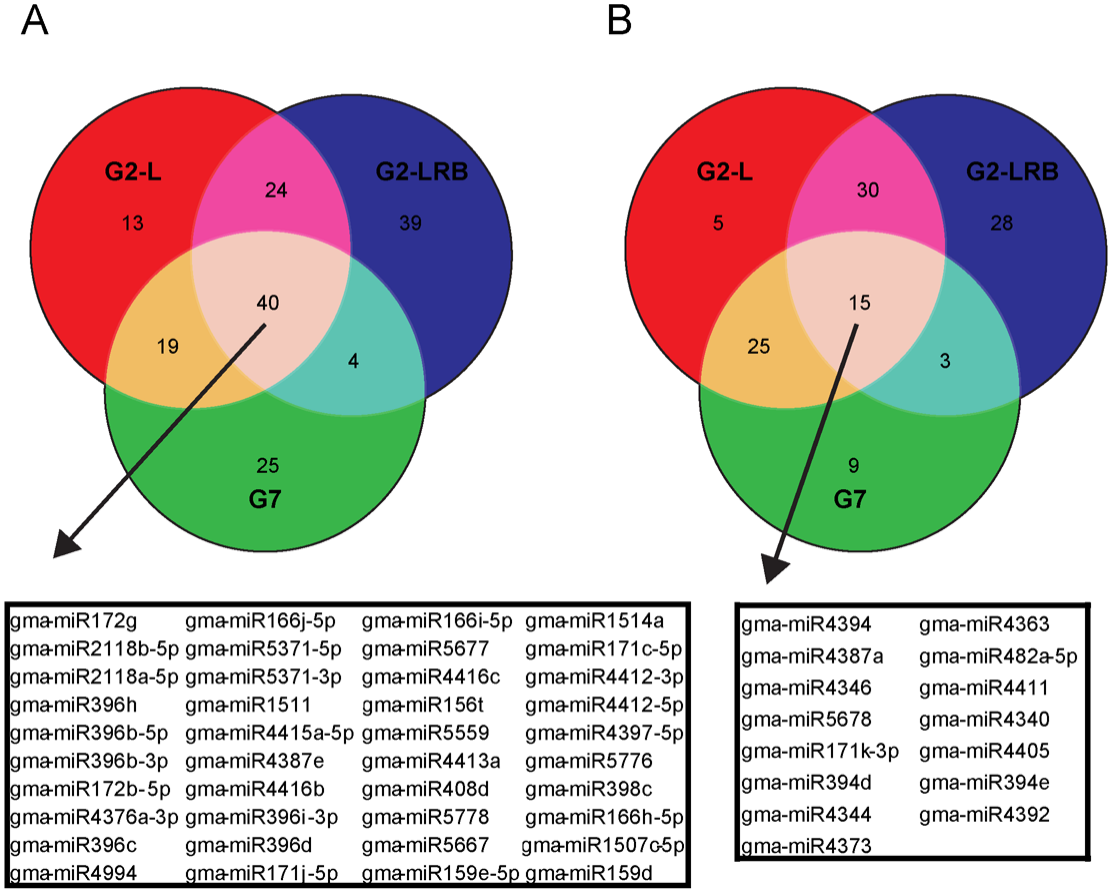
Comparison of differentially expressed miRNAs (DEMs) during infection by three SMV isolates. **(A)** Venn diagram of significantly upregulated DEMs among G2-L, G2-LRB and G7 infection relative to the mock-inoculated control. 40 DEMs were found to be upregulated during infection by all three SMV isolates. Box, the names of the 40 common DEMs. **(B)** Venn diagram of significantly downregulated DEMs among G2-L, G2-LRB and G7 infection in comparison with mock-inoculated control. 15 common DEMs were found to be downregulated during infection by three SMV isolates. Box, the names of 15 common DEMs that were significantly downregulated during infection by three SMV isolates are given.

To better understand the involvement of miRNA in SMV infection, miRNAs were identified that showed differential expression in soybean plants infected with one of three SMV isolates (G2-L, G2-LRB, and G7) compared to the mock control. A total of 253 soybean miRNAs, including both sense and antisense forms of miRNAs (-5p and -3p) which originated from the same predicted precursor and representing 97 families, were differentially regulated by at least a two-fold change [upregulated, Log_2_ (FC) ≥ +1.0; downregulated, Log_2_ (FC) ≤-1.0; quantile normalized raw counts normalized to million mapped miRNA reads] compared to mock-inoculated controls (S2 Table and Fig 1). 39 miRNAs were identified as being significantly upregulated unique to G2-LRB infection, compared to only 13 and 25 uniquely upregulated miRNAs associated with G2-L and G7 infections, respectively (Fig 2A). 28 downregulated miRNAs were unique to G2-LRB infection, in contrast to only 5 and 9 downregulated miRNAs in G2-L and G7 infections, respectively (Fig 2B). A total of 55 common miRNAs were differentially expressed in soybeans infected by all the three SMV isolates, including 40 upregulated (72.7%, Fig 2A) and 15 downregulated (27.3%, Fig 2B). Taken together these data suggest that while some soybean miRNAs are differentially expressed upon infection by a particular SMV isolate, there are a group of miRNAs that are commonly affected regardless of the isolate.

**Fig 3:**
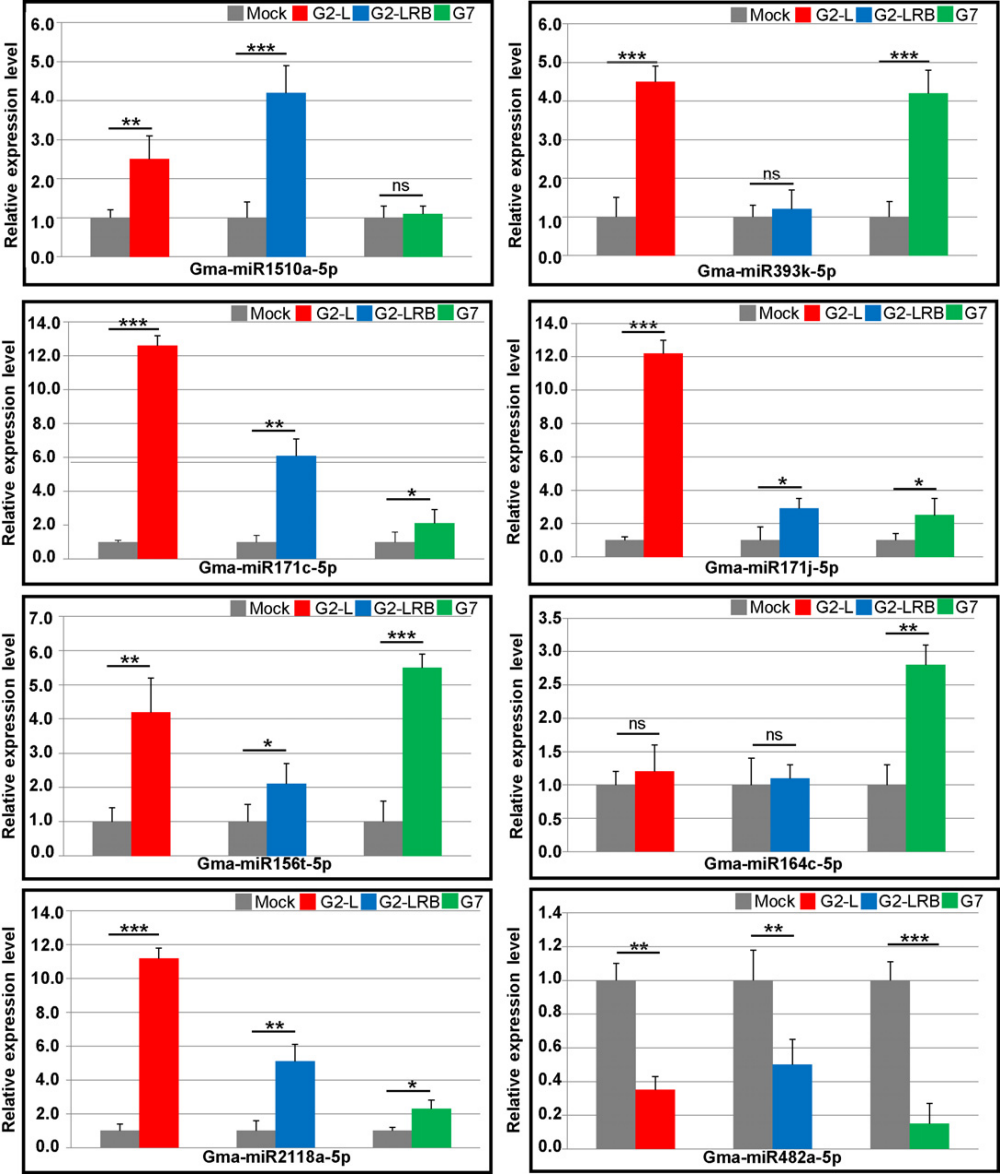
Stem-loop RT-qPCR analysis of miRNA expression in response to infection by three SMV isolates. Small RNAs fractions (≤ 200 nt) derived from SMV-infected and mock-inoculated plants were isolated at 14 dpi. Soybean 18S rRNA was used as an internal control. Error bars represent mean ± SD (standard deviation) and the data are averages from three biological replicates. Asterisks indicate statistically significant differences comparing with the mock control (student’s *t*-tests) (*p < 0.05, **p < 0.01, ***p < 0.001, ns, not significant).

**Fig 4:**
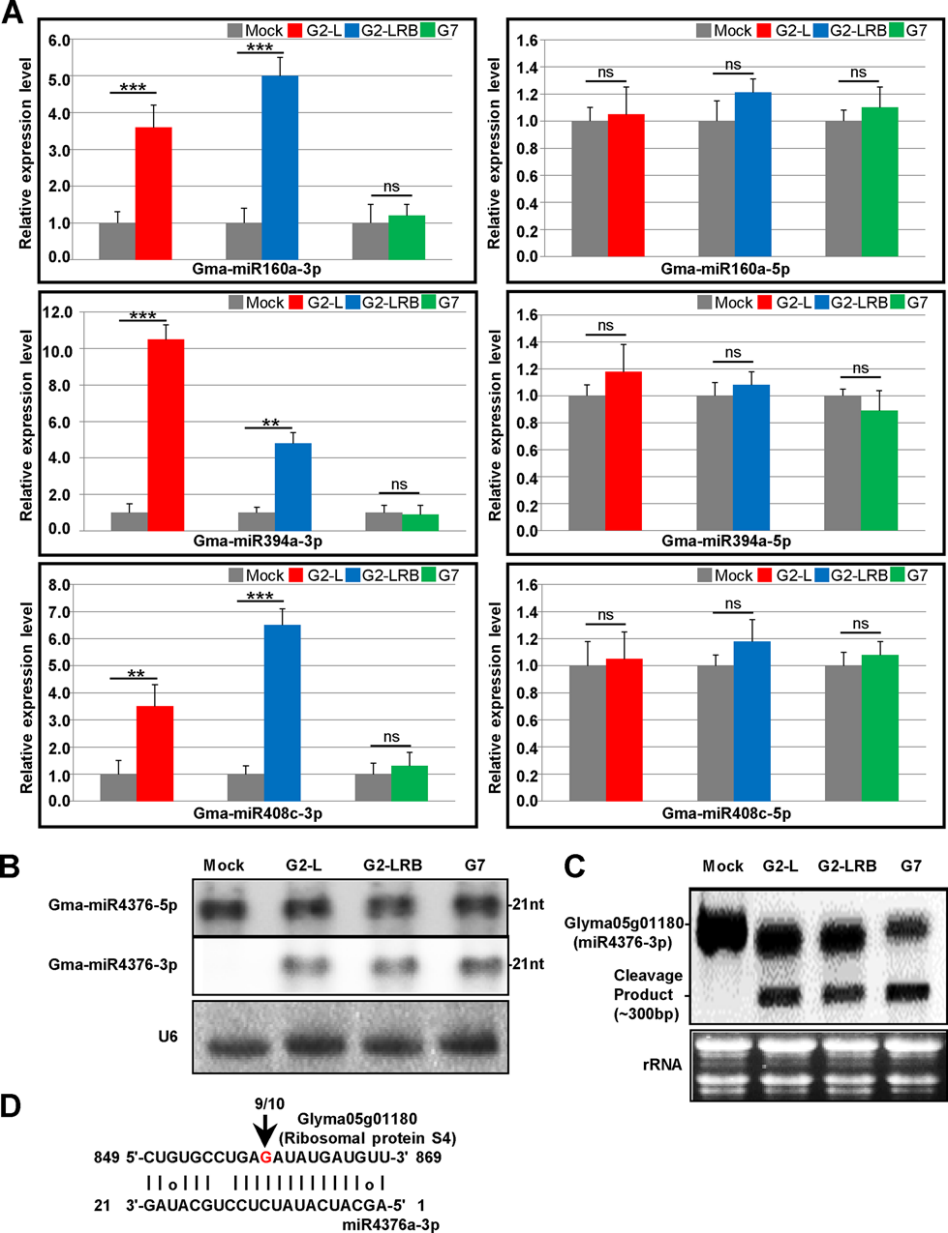
The enhanced accumulation of miRNA-3p in SMV-infected soybean plants. **(A)** Expression analysis of miRNA-5p and miRNA-3p in mock-inoculated and SMV-infected soybean plants at 14 dpi by stem-loop RT-qPCR analysis. Soybean 18S rRNA was used as an internal control. Error bars represent mean ± SD (standard deviation) and the data are averages from three biological replicates. Asterisks indicate statistically significant differences comparing with the mock control (student’s *t*-tests) (*p < 0.05, **p < 0.01, ***p < 0.001, ns, not significant). **(B)** Expression analysis of miRNA-43763p by Northern blots. Soybean U6 was used as an internal control to normalize miRNA accumulation. **(C)** Northern blot analysis of the expression of GLYMA05g01180, a predicted target of miR4376-3p. The rRNA stained with ethidium bromide was used as a loading control. **(D)** Mapping of the cleavage site in GLYMA05g01180 by RLM-5' RACE assay. The numbers above the arrows indicate the frequencies of sequenced RACE clones corresponding to the cleavage site.

We further looked into a handful of miRNAs that have been shown to be associated with infections by viruses or other pathogens in the literature. A previous study identified that three miRNAs (miR160, miR393 and miR1510) are involved in the soybean defense response to SMV infection (SC7 isolate) [35]. Our results confirmed this finding (S2 Table). Both miR160 and miR1510 were upregulated during G2-L and G2-LRB infections, and miR393 was significantly upregulated during G2-L and G7 infection (S2 Table and Fig 3). In *Arabidopsis*, miR171 is induced by *Turnip mosaic virus* (TuMV) infection and directs cleavage of several mRNAs coding for *Scarecrow-like* transcription factors [36]. MiR156 and miR164 can be induced by expressing viral silencing suppressors such as P1/HC-Pro encoded by TuMV [36] and p69 encoded by *Turnip yellow mosaic virus* (TYMV) [37]. Infection by any of the three SMV isolates upregulated members of both miR171 (miR171c/j-5p) and miR156 families (miR156t-5p) (Fig 3). However, miR164 (c/h/i-5p) was only induced by G7 infection (S2 Table and Fig 3). Several recent studies have revealed that the miR482/miR2118 superfamily in tomato, soybean and *Medicago truncatula*, targets numerous NB-LRRs defense genes at the conserved P-loop-encoding motif [31,38,39]. Moreover, in chickpea (*Cicer arietinum*), miR2118 is upregulated in response to wilt infection with the fungus *Fusarium oxysporum* [40]. However, it is downregulated after infection by the fungus *Verticillium dahliae* in cotton [41]. In tomato, miR482 is downregulated in leaves infected with CMV, *Turnip crinkle virus* (TCV) and *Tobacco rattle virus* (TRV) [38]. In this study, we found that miR2118a/b-5p was highly upregulated, whereas miR482a-5p was downregulated upon infections by any of the three isolates (Fig 3). We also checked antisense strand miRNAs (-3p) as many such miRNAs are accumulated at high levels in RSV-infected rice plants [24]. We found that SMV infection enhanced the accumulation of miRNAs-3p in some miRNA families, but not their corresponding miRNAs-5p. These included miRNAs-3p members of four miRNA families, i.e., miR160 (miR160a), miR171 (miR171b and miR171i), miR394 (miR394a and miR394b) and miR408 (miR408a and miR408c) (S2 Table and Fig 4A). For example, miR4376-3p was significantly induced by SMV infection (S2 Table), which was confirmed by Northern blot analysis (Fig 4B). Its corresponding miR4376-5p, however, did not show any obvious changes, compared to the mock control (Fig 4B). In tomato, miR4376 regulates the expression of an auto-inhibited Ca^2+^-ATPase (*ACA10*) during reproductive growth [42]. The *ACA10* transcript in soybean does not contain a miR4376 target site and it would be interesting to determine if this transcript is regulated by miR4376 in soybean. Interestingly, GLYMA05g01180, a ribosomal protein *S4* gene was predicted to be a target of miR4376-3p. We further found that GLYMA05g01180 expression was specifically downregulated by miR4376-3p and a cleavage product was detected in soybean plants infected by each of the three isolates (Fig 4C). The cleavage site in the miR4376-3p binding region was validated by 5' RACE (Fig 4D), suggesting a possible role for miR4376-3p in SMV infection. Therefore, like sense strand miRNAs, antisense strand miRNAs may also exert regulatory roles by directing the cleavage of their target mRNAs during viral infection.

### Prediction and Validation of the miRNAs Target by Degradome-Seq

To understand the potential regulatory roles of the detected SMV-responsive miRNAs, the target genes of all SMV-responsive miRNAs were predicted using Strand NGS (Strand Life Sciences, version 2.1). miRNA mapping to target genes was done via target prediction databases downloaded as annotations from soybean functional network (www.nclab.hit.edu.cn/SoyFN). A total of 325 target genes were identified under a standard set of criteria for target cleavage including a p-value cut-off of 0.05 (S3 Table). To further validate these potential miRNAs targets, four degradome libraries (G2-L, G2-LRB, G7 and mock control) were constructed and sequenced. This allowed for the large-scale examination of miRNA-guided cleavage products. A total of 31,190,930 degradome sequences (degradome-seq) were obtained and mapped to the soybean genome (GLYMA1, Ensembl), with approximately 56% (over 17 million) of sequences matching perfectly to the soybean genome. The degradome-seq sequences were also mapped to each soybean chromosome (S4 Table).

The identified targets were classified into five categories (0–4) based on the strength of degradome signal at the miRNA target sites [43]. A total of 3,145 transcripts were found to perfectly match the degradome tags. Under strict parameters, we identified 125 transcripts from four degradome libraries as potential cleavage targets by 26 miRNA families (105 members). 3, 15, 72 and 35 transcripts fell into categories 0–3, respectively (S5 Table). Diverse targets included *GLYMA11G20520*.*1* [Homeobox-leucine zipper (HD-ZIP) family protein/lipid-binding START domain-containing protein], *GLYMA15G09750*.*3* (auxin response factor 8), *GLYMA18G07890*.*2* (nuclear factor Y, subunit A1), *GLYMA01G18040*.*1* (GRAS family transcription factor), *GLYMA04G39741*.*1* (disease resistance protein) and *GLYMA05g01180*.*1* (ribosomal protein S4). These transcripts were respectively targeted by miR166i, miR167c, miR169a, miR171k, miR482a and miR4376, which was validated by RLM-5' RACE (S1 Fig). These experimental results provide additional support for the interactions of miRNAs and their target genes by providing supporting evidence for miRNA-guided transcript cleavage.

### Infection by Three Different SMV Isolates Leads to Common and Distinct Transcriptional Effects

**Fig 5:**
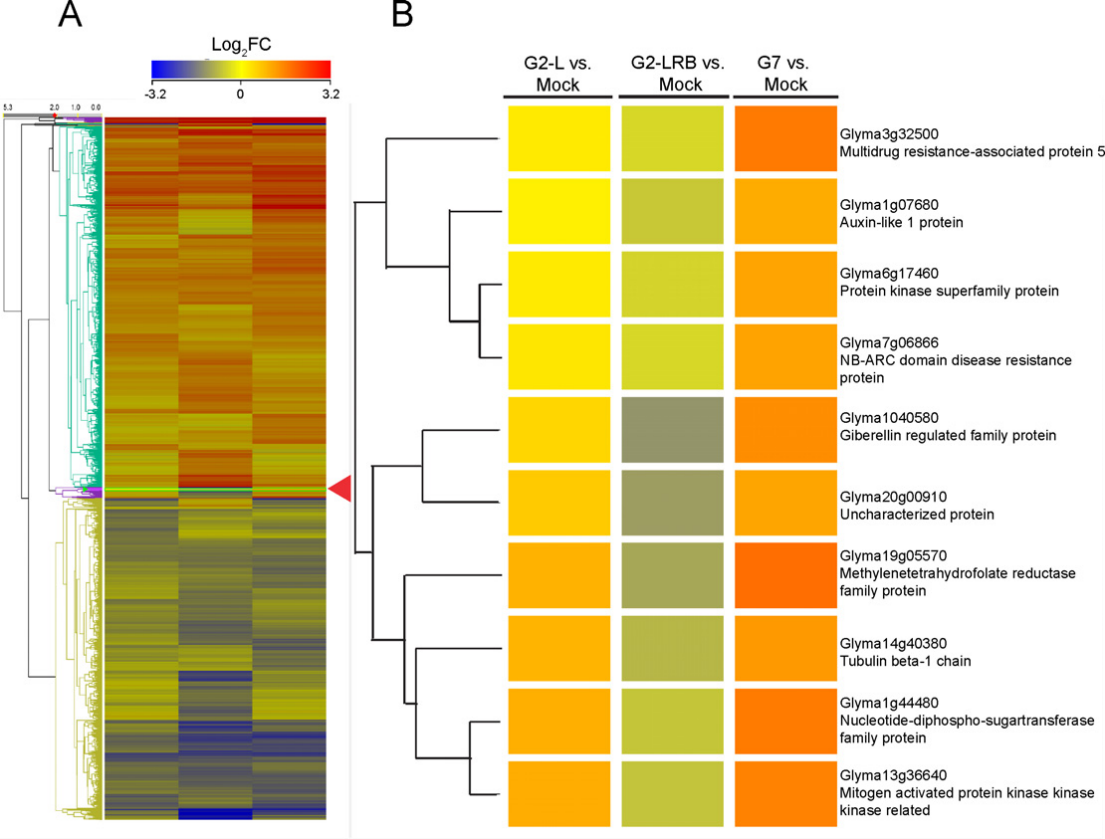
A hierarchical cluster analysis of global mRNA expression profiles in response to infection by different SMV isolates. **(A)** Heat map displaying 2679 differentially expressed genes (DEGs) in response to infections by three SMV isolates (G2-L, G2-LRB and G7) at 14 dpi. Heat map was generated using Strand NGS (http://www.strand-ngs.com/). **(B)** A detailed view of an exemplar branch of the heat map showing genes with differential responses following SMV isolate infection. Colors indicate the log_2_ fold changes (FC) with a ratio of SMV-infected sample versus the mock-inoculated control according to the average of the normalized signal values. Red colour denotes upregulation, while blue indicates downregulation. Read densities = RPKMs (Read count per kilobase of exon model per million reads); Normalized signal values = log2(RPKMs). Significance was determined by using a fold change threshold of at least 2.

**Fig 6:**
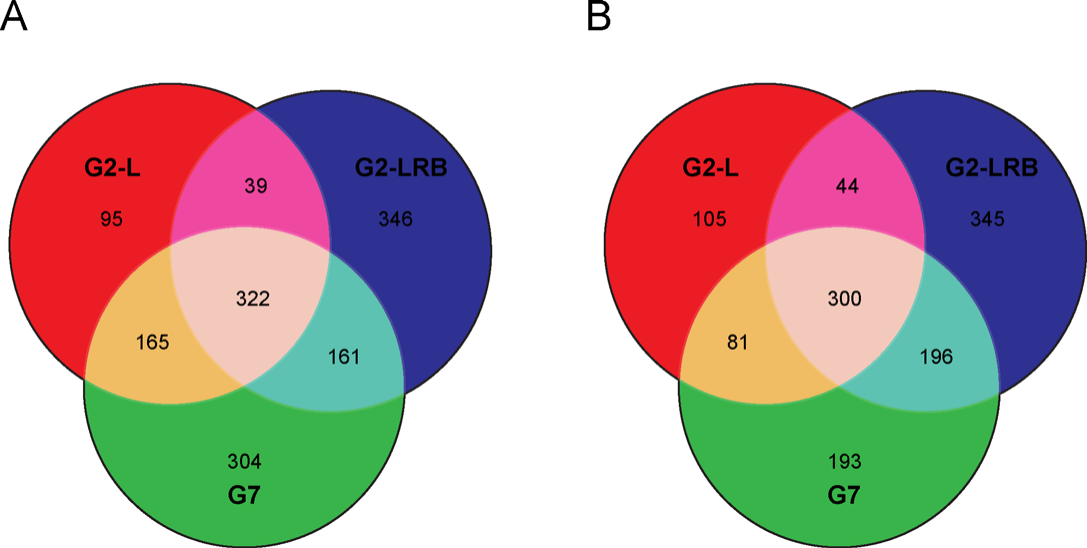
Comparison of differentially expressed genes (DEGs) in response to SMV infection. **(A)** Venn diagram of significantly upregulated DEGs among G2-L, G2-LRB and G7 infection relative to mock-inoculated control. **(B)** Venn diagram of significantly downregulated DEMs among G2-L, G2-LRB and G7 infection in comparison with the mock-inoculated control.

To understand the global transcriptional response during SMV infection and identify common targets, four transcriptome libraries (G2-L, G2-LRB, G7 and mock control) were constructed and sequenced. A total of 25,984,166 transcriptome sequences (RNA-seq) were obtained and mapped to the genome (S6 Table). About 76.5% (over 19 million) sequences were matched perfectly to the soybean genome, resulting in more than 4.8 million genome-matched RNA-seq sequences per library. For each library, over 2.8 million sequences were mapped to the soybean transcriptome (Ensembl) and more than 1.4 million paired reads aligned to the same transcripts. The distribution of reads in each soybean chromosome is shown in S6 Table. Differentially expressed genes (DEGs) were identified between SMV infected plants and a mock-inoculated control. A total of 2679 genes including 7 new genes showed differential expression of at least a two-fold change [Upregulated, Log_2_ (FC) ≥ +1.0; Downregulated, Log_2_ (FC) < = -1.0; quantile normalized raw counts used to generate RPKMs (Read count per kilobase of exon model per million reads)] in response to infection by one of the three SMV isolates (Fig 5 and S7 Table). 322 common DEGs were upregulated during infection with all three SMV isolates. 346 and 304 DEGs were significantly upregulated unique to G2-LRB and G7 infection, respectively, and 95 DEGs were associated only with G2-L infection (Fig 6A). 300 common DEGs were found to be downregulated during infection with all three SMV isolates. 345 downregulated DEGs were identified unique to G2-LRB infection in contrast to only 193 and 105 downregulated DEGs unique to G7 and G2-L infections, respectively (Fig 6B).

**Fig 7:**
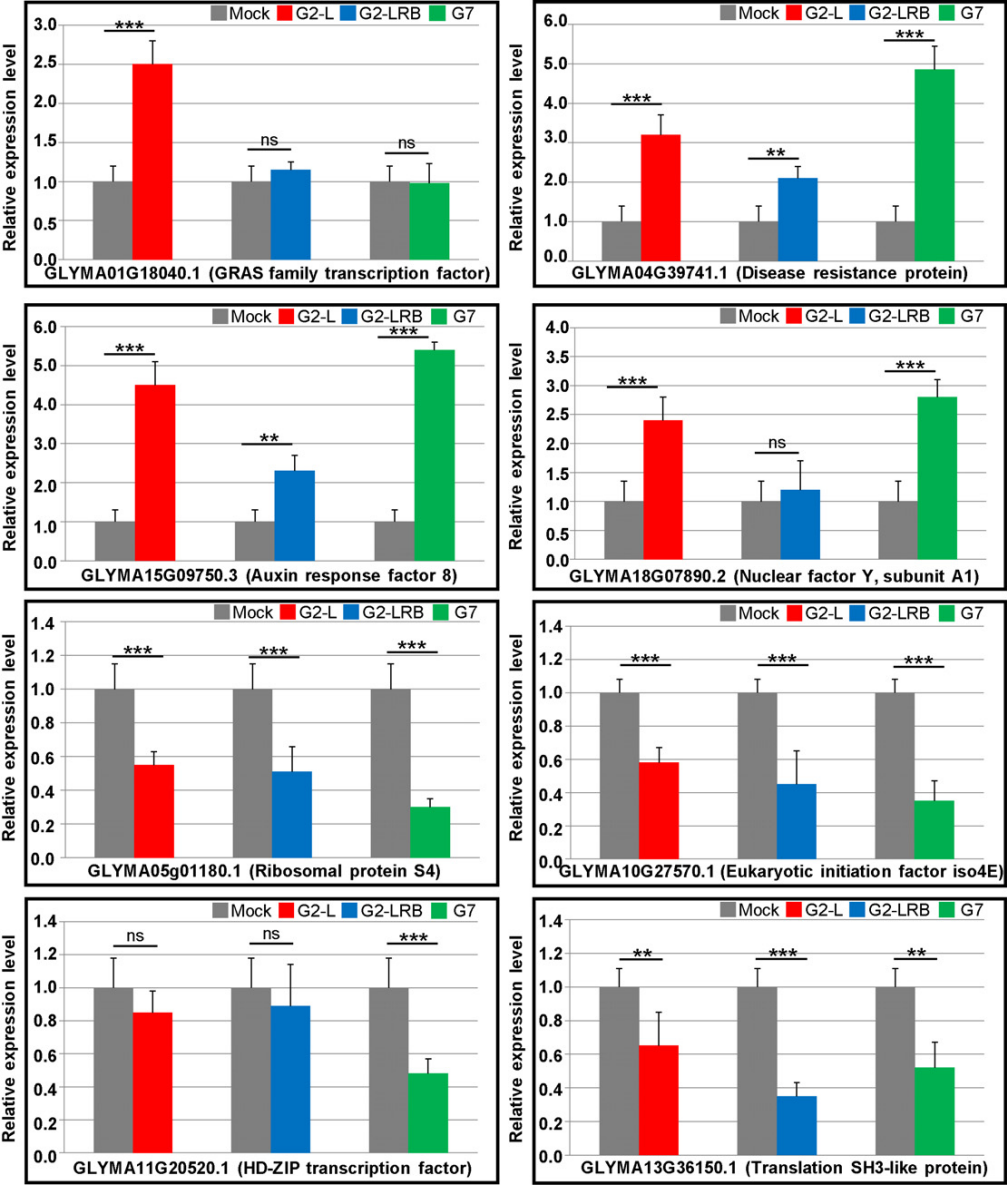
RT-qPCR validation of the differentially expressed genes (DEGs) in response to SMV infections with three isolates. Based on the results from RNA-seq combined with sRNA-seq and degradome-seq analysis, 8 DEGs that supposedly represented the majority of DEGs in response to SMV infection at 14 dpi were selected for validation by RT-qPCR analysis. The soybean *Actin* (*GmACT11*) gene was used as an internal control. Error bars represent mean ± SD (standard deviation) and the data are averages from three biological replicates. Asterisks indicate statistically significant differences comparing with the mock control (student’s *t*-tests) (*p < 0.05, **p < 0.01, ***p < 0.001, ns, not significant).

To validate these results, 8 DEGs (4 upregulated and 4 downregulated) were subjected to RT-qPCR analysis, including *GLYMA01G18040*.*1* (GRAS family transcription factor), *GLYMA04G39741*.*1* [Disease resistance protein (TIR-NBS-LRR class) family], *GLYMA15G09750*.*3* (Auxin response factor 8), *GLYMA18G07890*.*2* (Nuclear factor Y, subunit A1), *GLYMA05g01180*.*1* (Ribosomal protein S4), *GLYMA10G27570*.*1* (Eukaryotic initiation factor 4E protein, eIF4e), *GLYMA11G20520*.*1* (HD-ZIP family protein) and *GLYMA13G36150*.*1* (Translation protein SH3-like family protein). Overall, RT-qPCR data seemed largely similar to those obtained with RNA-seq analysis (Fig 7). *GLYMA01G18040*.*1*, a GRAS family transcription factor which was considered for its involvement in plant defence responses, was specifically induced by G2-L infection but not by G2-LRB and G7 infection. *GLYMA11G20520*.*1*, a HD-ZIP family gene which was thought to be involved in transcriptional regulation of a pathogen defense-related gene, was downregulated only by G7 infection. Interestingly, a plant defense-associated gene (*GLYMA04G39741*.*1*, TIR-NBS-LRR class) was upregulated, but a soybean eIF4e gene (*GLYMA10G27570*.*1*) which is critical for virus infection, was downregulated during SMV infection by all the three isolates. These analyses confirmed that host plants had different transcriptional responses depending on the infecting SMV strain, supporting that a series of host transcripts are specifically and significantly associated with SMV infection by different isolates.

**Fig 8:**
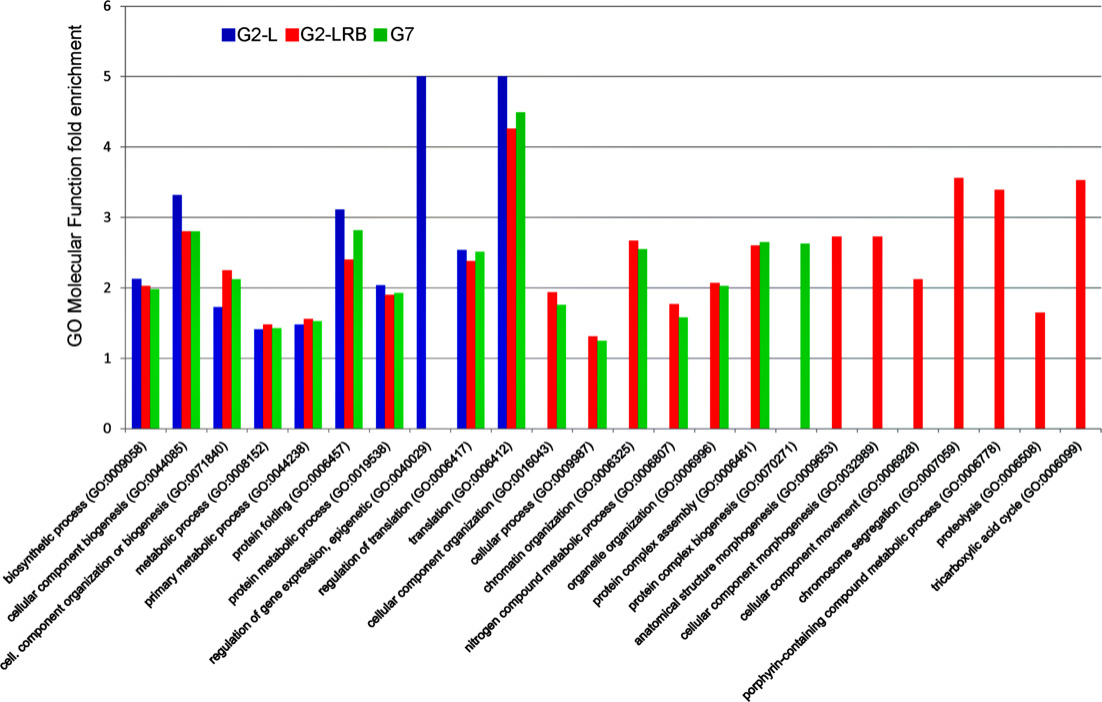
GO molecular function fold enrichment in DEGs following SMV infection. DEG responsive to infection with SMV isolates G2-L, G2-LRB and G7 were analyzed for enrichment of molecular function terms using the PANTHER classification system (pantherdb.org). All significant enrichments are shown (p<0.05, Bonferonni corrected).

To explore the biological relevance of 2679 DEGs in response to SMV infection, Gene Ontology (GO) and enrichment analysis were performed on the DEGs using the GO database (Soybase). Genes with predicted roles in immunity, cell division, apoptosis and DNA repair were significantly (corrected P <0.05) overrepresented/underrepresented under viral infection conditions (S7 Table). According to the enrichment analysis, 71 DEGs were involved in the defence response to plant pathogens (S8 Table). These include 52 *NB-LRR* genes, 10 genes encoding oxidative stress associated proteins such as lipoxygenase,respiratory burst oxidase, genes encoding disease resistance-responsive protein such as dirigent-like protein, MLP-like protein 423, glucan synthase-like 5 (GSL5) and syntaxin of plants 121(SYP121) (S8 Table). To compare the effects of infection by the three isolates on molecular function terms a GO enrichment was performed on the DEGs responsive to G2-L, G2-LRB, and G7 infection. G2-LRB infection resulted in a significant enrichment of 22 GO categories, including 7 uniquely enriched categories (Fig 8, p<0.05, Bonferroni corrected). G7 infection had 16 enriched terms with GO:0070271 (protein complex biogenesis) unique to the isolate. G2-L infection had the fewest enriched terms (10) with one unique term (Fig 8). Nine terms were commonly enriched by infection with all isolates including translation (GO:0006412), regulation of translation (GO:0006417), protein folding (GO:0006457) and primary metabolic process (GO:0044238). Together these results suggest that while the three SMV isolates induce the varying transcriptional responses, genes related to protein synthesis/modification and metabolism are commonly responsive regardless of SMV isolate.

### Constructing Complex Regulatory Networks Using miRNA and Transcriptome Data

**Fig 9:**
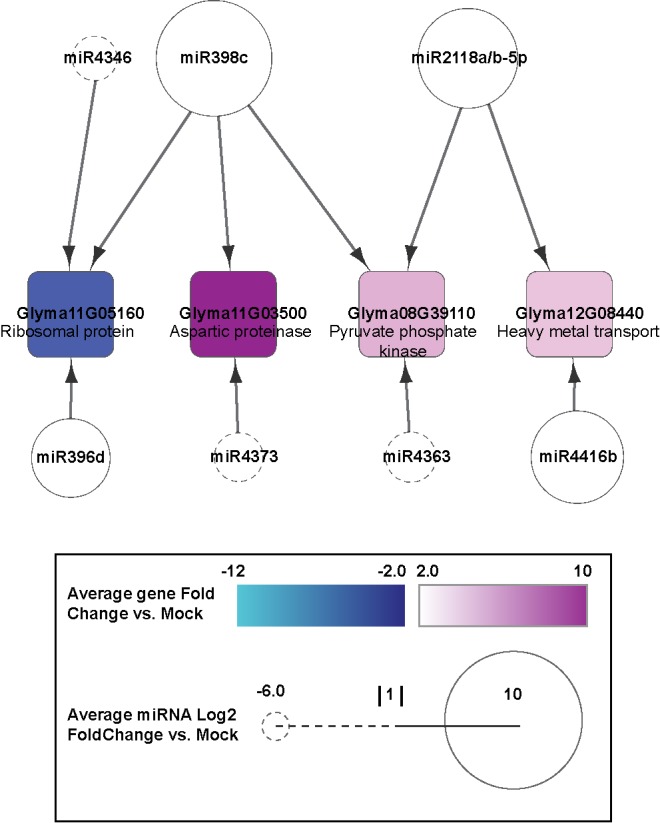
Regulatory connections of miRNAs and their target genes shared in infections by all the three SMV isolates. miRNAs are shown as circles. Negative Log_2_ fold change is represented by a dashed line when negative, and a solid line when positive. Target genes are represented by squares. Expression fold change >2 ranges from cyan to dark blue when negative, and white to magenta when positive.

In order to depict the complex relationship between miRNA regulation and transcriptional response to SMV infection with different isolates, the predicted interaction of miRNA-mRNA was visualized as networks using the RNA-seq, small RNA-seq and dergadome-seq data. 2679 genes and 253 miRNAs that exhibited at least a two-fold change [FC≥|2| or Log_2_ (FC)≥|1|] in expression in response to either G7, G2-L or G2-LRB infection were used in the analysis. miRNA targeting prediction identified 325 target genes. Of these, 37 were found to have at least a two-fold change in expression in response to infection by at least one of the three SMV isolates. These were then filtered against the degradome data set. The inclusion of the degradome data while not definitive of direct miRNA/mRNA interaction provided further support for the regulation of the targeted gene by their respective miRNAs. miRNAs were designated as “sources” and the genes they are predicted to regulate as “targets”. In network nomenclature an edge is a link or interaction between vertices (or nodes). In this case miRNAs and their targets comprise the nodes of the network and the predicted regulation of a target gene by a miRNA, the edges. Typically, arrowheads at the ends of edges denote positive interaction (upregulation) and the effects of miRNA are generally negative, arrowheads were used here to chiefly denote regulatory hierarchy and only potential regulatory interactions (S2 and S3 Figs and Fig 9). Infection by the G2-LRB isolate resulted in the largest regulatory network, comprising 84 nodes and 136 edges. Infection by the G2-L isolate produced a smaller network of 63 nodes and 113 edges, while G7 infection yielded a regulatory network consisting of 62 nodes and 78 edges. To examine the differences in the regulatory effects of infection by the three different isolates, miRNA/target gene interactions that were unique to infection by each isolate were identified. Similar to the trend observed previously, G2-LRB had the largest unique infection-triggered network (79 nodes, 101 edges) and contained some notable target genes. *GLYMA10g27570* is highly similar to and is very likely an orthologue of an isoform of the *Arabidopsis thaliana* EUKARYOTIC TRANSLATION INITIATION FACTOR 4E protein (eIFiso4E) (S2 Fig and S9 Table). *GLYMA14g00880* shares high similarity to the *Arabidopsis thaliana* SUPRESSOR OF AUXIN RESISTANCE 3 (SUP3) which is a nucleoporin shown to play a role in pathogen defense (Roth and Wiermer, 2012; Wiermer *et al*., 2012). The G2-L unique responsive network (45 nodes, 65 edges) also included a number of genes with potential roles in protein synthesis and modification as well as three target genes that may encode zinc finger proteins, *GLYMA11G07930*, *GLYMA14G01930*, and *GLYMA09G31470*. Similar to what was found in the G2-LRB network, the miRNA319 family supplied many edges to the G2-L network (S3 Fig and S9 Table). The unique regulatory network that arose in response to G7 infection was the sparsest (43 nodes, 51 edges) but similarly appeared to involve a number of target genes related to protein synthesis and modification (S3 Fig and S9 Table). A variety of genes involved in DNA binding and cell wall modification were also found to be uniquely induced by all the three SMV isolates (S10 Table).

### A Small Regulatory Network Common to Infection by All the Three SMV Isolates

In order to determine whether there are common regulatory responses to different SMV isolates, miRNA/gene interactions common to G2-LRB, G2-L, and G7 were analyzed. The network common to all SMV isolates comprised 12 nodes and 12 edges. GO analysis and comparison to related species revealed that *GLYMA11g05160* and *GLYMA11G03500* likely encode a 60S ribosomal protein and an aspartic proteinase, respectively. *GLYMA08G39100* may encode a pyruvate phosphatase and *GLYMA12G08440* is likely involved in heavy metal transport (Fig 9 and S9 Table). *GLYMA11g05160* was downregulated 3.2 fold, while *GLYMA11G03500*, *GLYMA08G39110*, and *GLYMA12G08440* were upregulated 11.1, 3.1, and 2.4 fold, respectively, in response to infection by any of the SMV isolates compared to mock treated plants. Three miRNAs take up noticeable roles in the network. Mir398c was upregulated 11.6 fold and targeted *GLYMA11G05160*, *GLYMA11G03500*, and *GLYMA08G39110*. miR2118a-5p and miR2118b-5p were both upregulated 8 fold and targeted *GLYMA08G39110* and *GLYMA12G08440*.

## Discussion

In this work, we profiled miRNAs in soybean infected by three SMV isolates using sRNA-seq and identified 55 DEMs (40 upregulated and 15 downregulated) in response to SMV infections regardless of isolates as well DEMS unique to infection by each isolate (Fig 2). G2-LRB infection resulted in the highest number of unique DEMs (39 upregulated and 28 downregulated), suggesting that G2-LRB strain resulted in the enrichment of a larger number of DEMs (S2 Table). Some miRNAs (miR5770a/b, miR5037c and miR862b) were found to be significantly upregulated only by G2-LRB infection, but not by G2-L and G7 infection. In addition, members of the widely conserved miR390 family (miR390a/b/c/d/f/g) that triggers tasiRNA biogenesis for an auxin-responsive regulatory network [44–46], were significantly downregulated only during G2-LRB infection (S2 Table). According to SL-RT-qPCR analysis, the accumulation of some antisense miRNAs-3p (miR160a-3p, miR394a-3p and miR408c-3p) were specifically enhanced during G2-L and G2-LRB infection, whereas their corresponding miRNAs-5p did not show any obvious changes compared to the mock control (Fig 4A). The miR393k-5p was highly induced by G2-L and G7 infection, but not by G2-LRB (Fig 3). Furthermore, the accumulation of miR171j-5p and miR164c-5p were profoundly increased only during G2-L and G7 infection, respectively (Fig 3). An in-depth analysis of more than 31 million degradome reads from four PARE libraries resulted in identification of 125 transcripts targeted by 26 miRNA families (105 members) under a strict filter (S5 Table). Among these, some were found to be involved in regulation of transcription such as the HD-ZIP family transcription factor (*GLYMA11G20520*.*1*), GRAS family transcription factor (*GLYMA01G18040*.*1*) and nuclear transcription factor Y (*GLYMA18G07890*.*2*). Some plant defense-related genes such as leucine-rich repeat protein kinase family genes (*GLYMA04G39741*.*1* and *GLYMA13G27540*.*1*) and TIR-NBS-LRR resistance genes (*GLYMA04G39741*.*1*) were also identified in the degradome-seq analysis (S5 Table). Moreover, 6 transcripts targeted by miR166i, miR167c, miR169a, miR171k, miR482a and miR4376 respectively were selected for experimental validation by RLM-5' RACE assay (S1 Fig). Overall, these observations support the accuracy of the sRNA-seq and degradome-seq analysis. Although the roles of common and strain/isolate-specific DEMs requires further investigation, which is beyond the scope of the current study, our results suggest that in addition to shared biological process occurring in infection by three different isolates, the strain/isolate-specific DEMs signatures are associated with the distinct pathogenesis of SMV strains/isolates.

High-throughput transcriptome-seq provides a powerful approach to identify global transcriptional responses to virus infection. Our transcriptome analysis of the host transcriptional response to SMV infection demonstrated that virus infection alters the expression of numerous DEGs, and these DEGs show transcriptional responses specific to SMV infection with different strains. The analysis of more than 25 million of RNA-seq sequences from four transcriptome libraries resulted in 2679 DEGs responsive to SMV infection with three isolates (S7 Table). Among these, 322 upregulated and 300 downregulated DEGs have common expression during SMV infection with all three isolates in comparison with the mock-inoculated control (Fig 6A and 6B). In turn, there were 346 upregulated and 345 downregulated DEGs only affected during G2-LRB infection but not during G2-L and G7 infection, suggesting that G2-LRB infection induces a more vigorous host response associated with higher number of DEGs. Of the 2679 DEGs, 71 DEGs including 52 *NB-LRR* genes and 10 oxidative stress associated encoding genes are involved in plant defense response to pathogens using GO enrichment analysis (S8 Table). Based on the results from RT-qPCR analysis, a GRAS family transcription factor (*GLYMA01G18040*.*1*) was highly induced only by G2-L infection, but not by G2-LRB and G7 infection, and a HD-ZIP family gene (*GLYMA11G20520*.*1*) was downregulated only by G7 infection. In addition, a soybean translation initiation factor eIF4e (*GLYMA10G27570*.*1*) considered to be required for virus infection was downregulated during SMV infection with any isolate (Fig 7). A ribosomal protein S4 gene (*GLYMA05g01180*) was identified as a novel target by miR4376-3p in this study and was downregulated by infections with any of the isolates (Fig 4C and 4D and Fig 7), suggesting that the interaction plays a key role in SMV infection. An enrichment analysis of GO molecular function provided further support for the heightened response initiated by G2-LRB infection. The G2-LRB isolate had the most unique enriched terms as well as the most enriched terms overall (Fig 8). All isolates had uniquely enriched terms highlighting the differences in the plants response to their infection. The nine enriched terms represented by the DEGs commonly after infection by any of the isolates points to protein synthesis and modification as the common class of genes affected. Translation (GO:0006412), regulation of translation (GO:006417), biosynthetic process (GO:0009058) and cellular component biogenesis (GO:0044085) enrichment likely point to viral hijacking of the cellular machinery for replication as well as the plants own defense response. Enrichment of metabolic process (GO:0008152) and primary metabolic process (GO:0044238) terms reflect the importance of energy management during viral infection. To manage the increase in energy required due to the upregulation of genes involved in disease defense and protein synthesis, the plant may need to finely tune its metabolic output. These data identified a validated set of transcripts associated and significantly responsive to infection by three different SMV isolates and point to the unique as well as common responses infections triggered by the host. Together they provided a resource of novel targets for further studies aiming to further explore the pathologies of these three SMV isolates as well as universal plant responses to SMV infection.

Having obtained RNAseq, small RNAseq and degradome data from soybean in response to SMV infection allows us to construct complex regulatory networks that are activated following exposure to the virus. However, these networks hinge on computationally predicted miRNA targeting, degradome-seq does not confirm direct miRNA/mRNA interaction and well characterized soybean proteins are rare. Despite these limitations these networks they can nonetheless provide valuable insight for guiding future research by highlighting possible shared and unique regulatory links between miRNAs and their target mRNAs. Whether considering the total or unique networks activated by infection, the G2-LRB strain consistently resulted in more robust networks with more nodes and edges. Additionally, a likely soybean orthologue of the *Arabidopsis* eIFiso4E protein appears to be a target during infection by G2-LRB strains [47]. Currently, most of the characterized recessive resistance genes to viruses encode eukaryotic translation initiation factors including those in lettuce (*Lactuca sativa*), melon (*Cucumis melo*), tomato (*Solanum lycopersicum*), pepper (*Capsicum annuum*), barley (*Hordeum vulgare*), pea (*Pisum sativum*) and rice (*Oryza sativa*) [47]. The large observed network is consistent with the relatively higher numbers of DEGs and DEMs observed following G2-LRB infection compared to the other two isolates and is likely due to the relative higher pathogenicity of the isolate. Previous work has revealed that a single amino acid substitution in the P3 protein of G2-LRB enables it to break SMV *Rsv4* resistance [13]. The G2-L isolate lacks this mutation and is an avirulent determinant for *Rsv4* [11]. The SMV G7 isolate, however, is virulent in the *Rsv1* soybean genotype, which confers extreme resistance against most SMV strains [48].

Genome wide analyses of miRNAs and their targets can yield thousands of significantly dynamic loci. Generating hypotheses from such complex data is a difficult task. Constructing regulatory networks using miRNA and target data allowed for data distillation and easier interpretation. In order to further narrow the candidate list, miRNAs and target transcripts responsive to infections by all isolates were identified. The resulting network was surprisingly simple (Fig 9). The shared regulatory network activated in response to all SMV strains found a majority of targets likely involved in protein synthesis and modification. A previous study analysing transcript abundance following SMV infection at 14 dpi similarly found a high number of transcripts predicted to have a role in protein synthesis and translation [14]. These nodes represent the most promising candidates for future research as they represent regulatory connections conserved in infections by any isolate. While these genes are not characterized in soybean, orthologues in model species such as *Arabidopsis* may provide a clue to their role in SMV infection. *GLYMA08G39110* is a likely ortholog of *AT1G10760* (*SEX1*) which encodes an α-glucan, water dikinase required for starch degradation [49]. Mutations in the *SEX1* gene affect transitory starch turnover and have a deep impact on plant development. In the null mutant, starch accumulates 5 times more than that in wild type plants. Moreover, mutant plants are massively compromised in growth [49,50]. The relationship between viral infection, metabolic changes and pyruvate kinases has been described previously [50–52]. SMV infection could actively promote starch hydrolysis to meet its reproductive energy demands. The aspartic protease *GLYMA11G03500* could also play an important role during SMV infection. In rice, an aspartic protease-reporter construct shows an increase in activity following inoculation with *Cucumber mosaic virus* (CMV). A knockout mutant is more susceptible to the virus [53]. In the network, 3 of the 4 four targets are upregulated. Typically miRNA have a negative effect of target transcript there is evidence suggesting that some miRNAs could upregulate gene expression in specific cell types and conditions with distinct transcripts and proteins [54–57]. There may also be additional regulatory factors beyond miRNAs involved in regulating these genes and the cumulative effect is represented here. However, these findings suggest potential key targets of SMV infection, both target genes as well as their miRNA regulators, specific to the three SMV isolates as well as ones common to all. As most of our identified targets are uncharacterized in soybean, this work identifies a wealth of opportunities for future research.

## Experimental Procedures

### Soybean Cultivar, Virus Strains, Inoculation and Detection

Soybean [*Glycine Max* (L.) Merr.] cultivar Williams 82 (susceptible) was planted and grown in a growth chamber under a 16 h light at 22°C and 8 h dark at 18°C. SMV infectious clones derived from SMV G2 (L and LRB isolates) [13] and G7 strain [58] were biolistically introduced into Williams 82 seedlings and the infected tissues were used as inoculum for further mechanical inoculation essentially as previously described [13,59]. Viral infections were monitored by visual observation of typical viral symptoms and RT-PCR analysis as previously described [28]. Soybean systemic leaf tissues of 15 infected plants from each treatment (inoculation by G2-L, G2-LRB, G7 or mock) were harvested and pooled for RNA extraction and library construction [14].

### Small RNA Library Construction, Sequencing and Data Analysis

Small RNA libraries were constructed as described previously with minor modifications [28,60]. For details on modifications see S1 Methods.

### Degradome Library Construction, Sequencing and Data Analysis

Degradome libraries were constructed as previously described with small modifications [31,61,62]. For details on modifications see S1 Methods.

### Transcriptome Library Construction, Sequencing and Data Analysis

Transcriptome libraries were constructed as previously described [63–65]. For details on modifications see S1 Methods.

### RNA Blot Analysis

Total RNA was extracted using TRIzol reagent (Invitrogen), and small RNA fractions (≤ 200 nt) were purified from total RNA using a mirVana miRNA isolation kit (Ambion). RNA blot for miRNAs detection was carried out with DIG-labeled RNA probes, which generated by *in vitro* transcription using a *mir*Vana™ miRNA probe construction kit (Ambion) with DIG-labeled UTP (Roche). For details see S1 Methods.

### Stem-Loop RT-qPCR

The stem-loop RT-qPCR assay was carried out as described previously [66,67]. Briefly, the reverse transcription was performed using TaqMan® MicroRNA Reverse Transcription Kit (Applied Biosystems) following the manufacturer's protocol with a stem-loop RT primer that binds to the 3΄ portion of the miRNAs. The RT product was amplified using the TaqMan® Universal PCR Master Mix (Applied Biosystems) with a miRNAs-specific forward primer and a universal reverse primer. Soybean 18S rRNA was used as an internal control. Primer sequences are included as a separate table in the S11 Table.

### RLM-5' RACE

RNA ligase-mediated 5' amplification of cDNA ends (RLM-5' RACE) was performed using the FirstChoice RLM-RACE Kit (Ambion) as previously described [68]. For details on modifications see S1 Methods.

### qRT-PCR

Total RNA was extracted from leaf tissues of mock-inoculated and SMV-infected plants at 14 dpi using TRIzol reagent (Invitrogen). One μg total RNA was reverse transcribed with Superscript III Reverse Transcriptase kit (Life) using gene specific reverse primer. qPCR was performed using the respective forward and reverse primer pairs as shown in the S11 Table. The soybean gene *Actin* (*GmACT11*) was used as an internal control. Three independent experiments (each including five soybean plants) were performed.

### Generating Interaction Networks of miRNA-mRNA

The differentially expressed genes (DEGs) demonstrating at least a two-fold change expression in comparison with mock-inoculated controls in response to SMV infection were used as “targets”. The differentially expressed miRNAs (DEMs) that showed at least a two-fold change expression in comparison with mock-inoculated controls in response to SMV infection were used as “sources”. The target genes of all SMV-responsive DEMs were predicted following the `Find Targeted Genes' step under the section `Results Interpretation' in the small RNA analysis workflow using the Strand NGS software (Strand Life Sciences, version 2.1) and the web tool psRNATarget [69]. Filtering based on expression changes and predicted targeting resulted in the genes that showed at least a two-fold change in expression in response to infection by one of the three isolates, were targeted by miRNA that also showed a change in expression of at least two-fold change. Targeted genes had to have corresponding degradome data to be included in the analysis. The above listed data was compiled and networks generated using Cytoscape 3.2.1[70].

## Supporting Information

S1 Fig Target plots (t-plots) of identified miRNA targets by degradome-seq. T-plots are shown in the top panel and the sequence alignments of miRNA and their targets are shown in the bottom panel for gma-miR169a, gma-miR166i-3p, gma-miR167c, gma-miR171k-3p, gma-miR4376-3p and gma-miR482a-3p, respectively. In the t-plots, the degradome sequence corresponding to the miRNA-directed cleaved transcript is represented by a red diamond and black arrowhead. The *X* axis indicates the nucleotide position on targeted transcript (nt, nucleotide). The *Y* axis indicates the normalised read abundance (TPM, transcripts per million) of cleaved transcript detected in degradome-seq. In the alignments, the vertical lines, missing lines and circles indicate matches, mismatches, and G:U wobble pairs, respectively. The black arrowheads (red colored nucleotide) above the target transcript indicate the cleavage site detected in the degradome-seq. The numbers of clones sequenced show the cleavage frequencies detected by 5′ RLM-RACE assay.
(TIF)

S2 Fig Regulatory connections of miRNAs and their target genes unique to G2-LRB infection. miRNAs are shown as circles. Negative Log_2_ fold change is represented by a dashed line when negative or a solid line when positive. Target genes are shown as squares. Expression fold change ≥2 ranges from cyan to dark blue when negative, and white to magenta when positive.
(TIF)

S3 Fig Regulatory connections of miRNAs and their target genes unique to infection by G2-L or G7. miRNAs are shown as circles. Negative Log_2_ fold change is represented by a dashed line when negative, and a solid line when positive. Target genes are displayed as squares. Expression fold change ≥2 ranges from cyan to dark blue when negative, and white to magenta when positive.
(TIF)

S1 Methods (DOCX)

S1 Table Summary of small RNAs (sRNA-Seq) library datasets from mock- and virus-infected soybean. (XLSX)

S2 Table List of differentially expressed miRNAs (DEMs) in response to infections by SMV G2-L, G2-LRB and G7 in soybean. (XLTX)

S3 Table List of predicted targets by SMV-responsive DEMs in soybean. (XLSX)

S4 Table Summary of PARE (Degradome-Seq) library datasets from mock- and virus-infected soybean. (XLSX)

S5 Table List of identified miRNA targets by degradome-seq in soybean. (XLSX)

S6 Table Summary of transcriptome (RNA-seq) library datasets from mock- and virus-infected soybean. (XLSX)

S7 Table List of differentially expressed genes (DEGs) in response to infections by SMV G2-L, G2-LRB and G7 in soybean. (XLSX)

S8 Table List of defense-related SMV-responsive DEGs in soybean. (XLSX)

S9 Table List of genes involved in the shared or unique regulatory network induced by SMV infection with three isolates in soybean. (XLSX)

S10 Table List of genes involved in DNA binding and cell wall modification uniquely induced by each SMV isolate in soybean. (XLSX)

S11 Table Primer sequences used in this study. (XLSX)
