# Correction: Genome-Wide Investigation Using sRNA-Seq, Degradome-Seq and Transcriptome-Seq Reveals Regulatory Networks of microRNAs and Their Target Genes in Soybean during *Soybean mosaic virus* Infection

**DOI:** 10.1371/journal.pone.0188140

**Published:** 2017-11-09

**Authors:** Hui Chen, Andrej Adam Arsovski, Kangfu Yu, Aiming Wang

S10 Table is a duplicate of S11 Table. Please view the correct S10 Table below.

## Supporting information

S10 Table List of genes involved in DNA binding and cell wall modification uniquely induced by each SMV isolate in soybean. (XLSX)
