# Supplementary figures and images for: Genome-Wide Investigation Using sRNA-Seq, Degradome-Seq and Transcriptome-Seq Reveals Regulatory Networks of microRNAs and Their Target Genes in Soybean during Soybean mosaic virus Infection
Source: PLoS One. 2016 Mar 10;11(3):e0150582. doi: 10.1371/journal.pone.0150582 (PMC4786119; doi:10.1371/journal.pone.0150582)

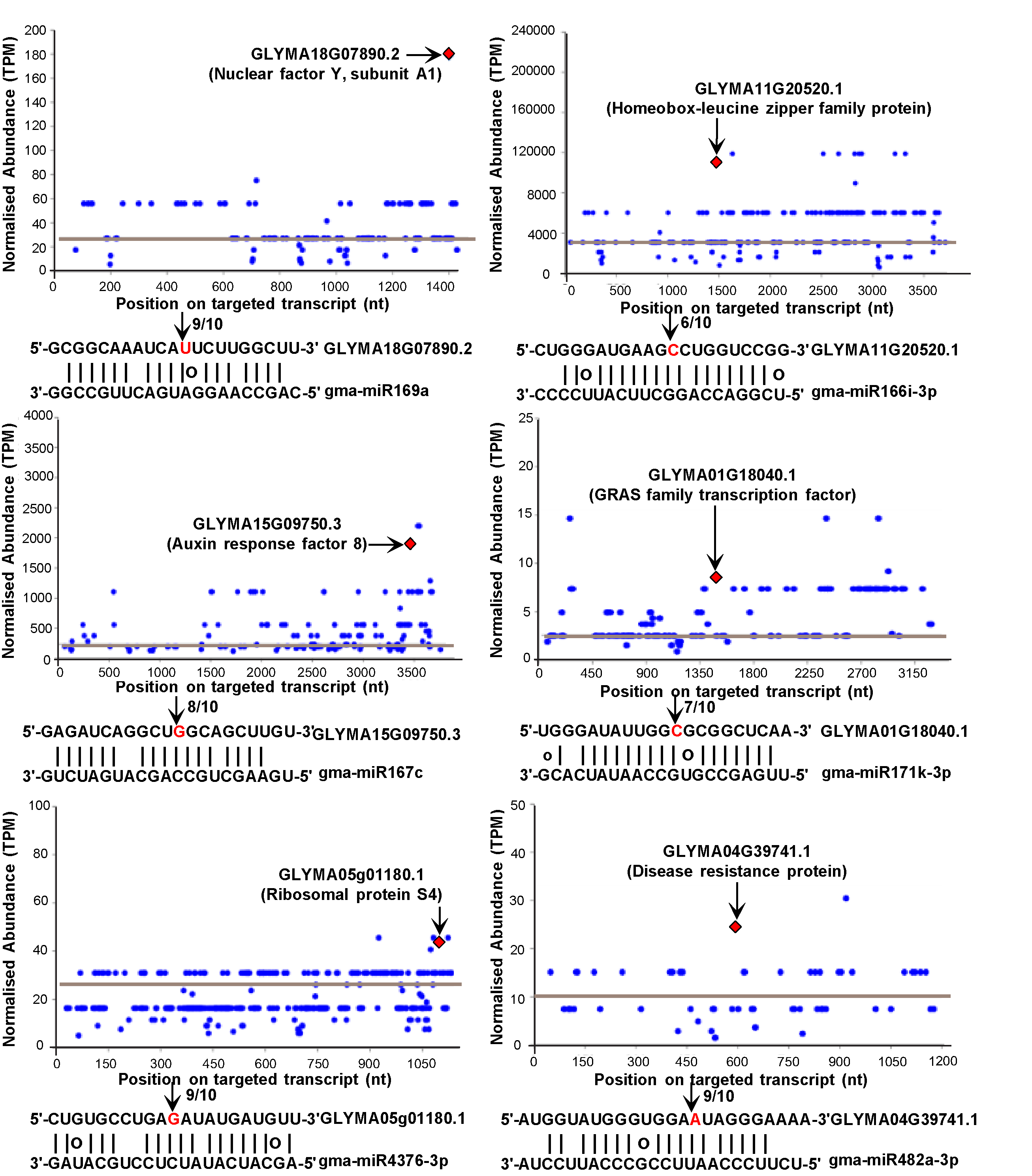

Supplement: S1 Fig — T-plots are shown in the top panel and the sequence alignments of miRNA and their targets are shown in the bottom panel for gma-miR169a, gma-miR166i-3p, gma-miR167c, gma-miR171k-3p, gma-miR4376-3p and gma-miR482a-3p, respectively. In the t-plots, the degradome sequence corresponding to the miRNA-directed cleaved transcript is represented by a red diamond and black arrowhead. The X axis indicates the nucleotide position on targeted transcript (nt, nucleotide). The Y axis indicates the normalised read abundance (TPM, transcripts per million) of cleaved transcript detected in degradome-seq. In the alignments, the vertical lines, missing lines and circles indicate matches, mismatches, and G:U wobble pairs, respectively. The black arrowheads (red colored nucleotide) above the target transcript indicate the cleavage site detected in the degradome-seq. The numbers of clones sequenced show the cleavage frequencies detected by 5′ RLM-RACE assay. (TIF) [file pone.0150582.s001.tif]

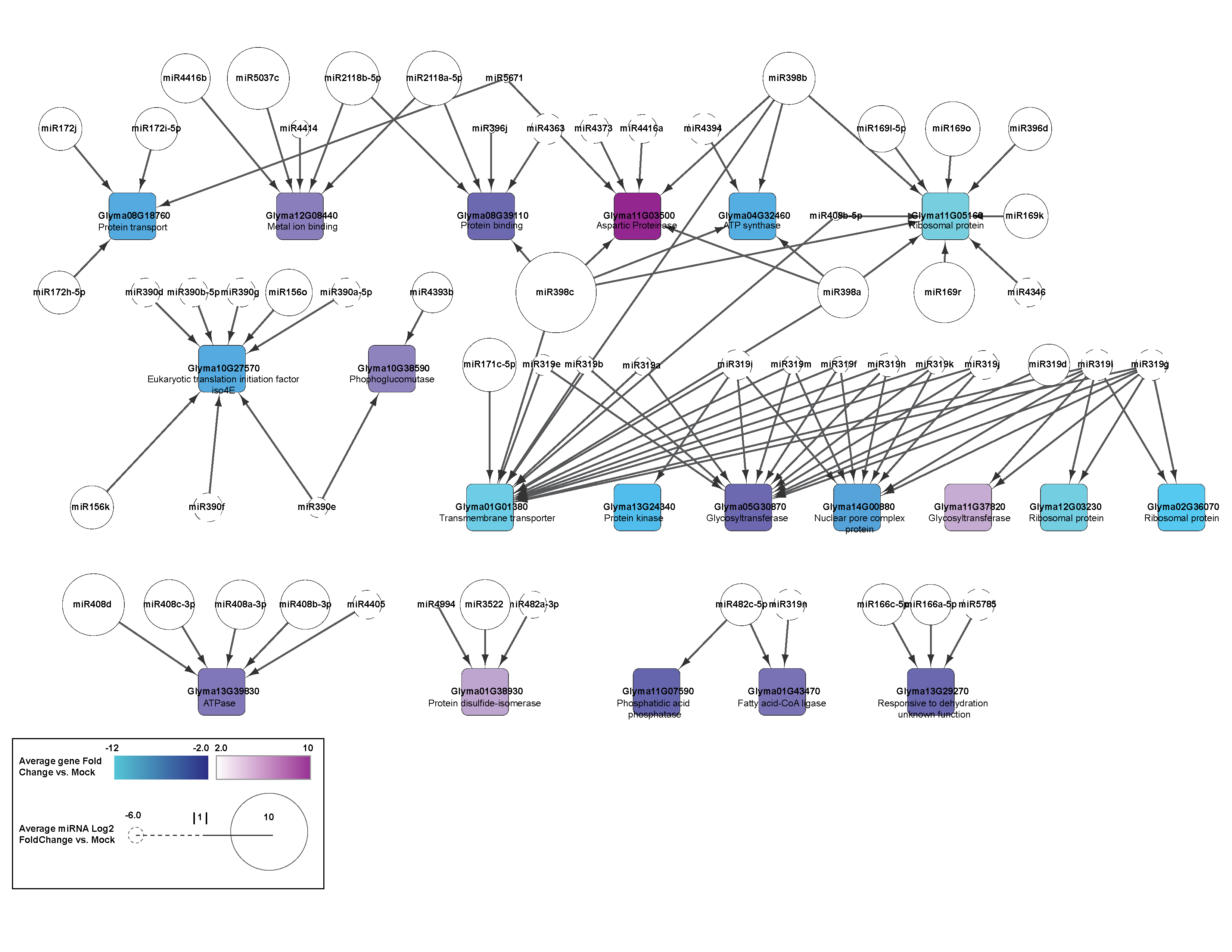

Supplement: S2 Fig — miRNAs are shown as circles. Negative Log2 fold change is represented by a dashed line when negative or a solid line when positive. Target genes are shown as squares. Expression fold change ≥2 ranges from cyan to dark blue when negative, and white to magenta when positive. (TIF) [file pone.0150582.s002.tif]

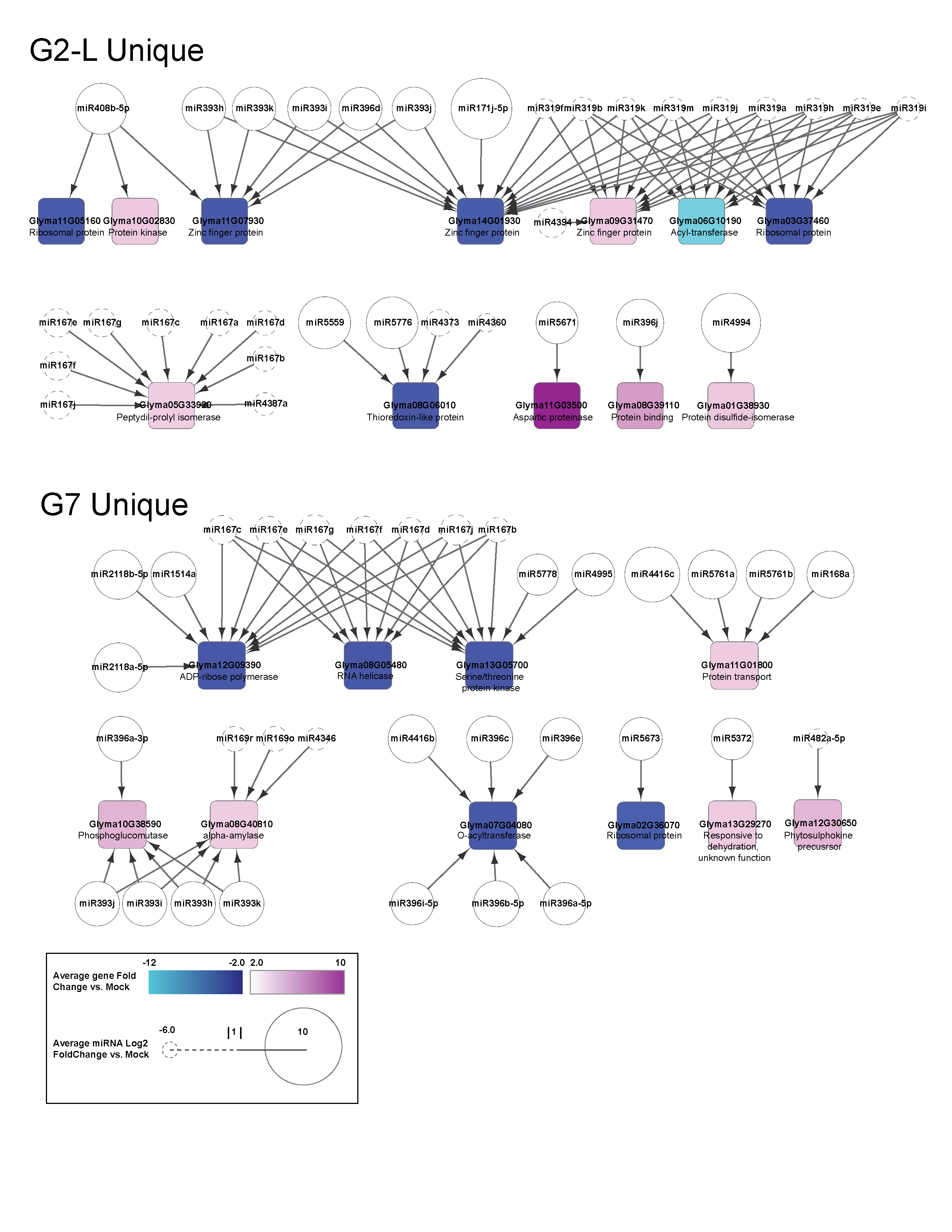

Supplement: S3 Fig — miRNAs are shown as circles. Negative Log2 fold change is represented by a dashed line when negative, and a solid line when positive. Target genes are displayed as squares. Expression fold change ≥2 ranges from cyan to dark blue when negative, and white to magenta when positive. (TIF) [file pone.0150582.s003.tif]
