# Supplementary material for: Genome-Wide Investigation Using sRNA-Seq, Degradome-Seq and Transcriptome-Seq Reveals Regulatory Networks of microRNAs and Their Target Genes in Soybean during Soybean mosaic virus Infection
Source: PLoS One. 2016 Mar 10;11(3):e0150582. doi: 10.1371/journal.pone.0150582 (PMC4786119; doi:10.1371/journal.pone.0150582)
Supplement: S1 Methods — (DOCX) [file pone.0150582.s004.docx]

**Supplemental Materials and Methods**

#### Small RNA library construction, sequencing and data analysis

#### Small RNA fractions (≤ 200 nt) derived from SMV-infected and mock-inoculated plants were isolated using a mirVana miRNA isolation kit (Ambion). The size range and quantification of small RNA fractions were assessed using a small RNA kit on the Bioanalyzer 2100 system (Agilent). Small RNA library construction was performed using TruSeq^TM^ small RNA sample prep kit (Illumina) following the manufacturer's instructions. The quality of cDNA libraries was assessed using a high sensitivity DNA chip on an Agilent 2100 Bioanalyzer (Agilent) and a library quantification qPCR kit (KAPA Biosystems) on a CFX96 real-time PCR detection system (Bio-Rad). Deep sequencing was done using the Illumina Miseq platform.

#### For sRNA-seq analysis, low quality reads were removed and the adapter sequences were trimmed. The resulting sequences were mapped to the *Glycine max* reference genome (Glyma1, Ensembl) [4]. Alignment to known *Glycine max* miRNAs (miRBase 21) was done using the Strand NGS software (Strand Life Sciences, version 2.1) according to the small RNA alignment and small RNA analysis pipeline using standard parameters. Reads showing 100% match to the genome were used for further analysis. MiRNA sequence data was quantile normalized. This was applied to all genes to obtain a scaling factor for each sample. The scaling factor is computed as the ratio of the sum of raw counts to the sum of normalized counts. This scaling factor is then used to arrive at the final normalized counts, which are obtained by dividing the raw counts for each gene with the scaling factor of the respective sample. These normalized counts are then log transformed to arrive at the final normalized signal values. Normalized signal values are displayed in the Heatmap.

#### Degradome library construction, sequencing and data analysis

#### Total RNA was extracted using a mirVana miRNA isolation kit (Ambion) and Poly (A)^+^ RNA was isolated using the NucleoTrap^@^ mRNA purification kits (Machery-Nagel) following the supplier’s manual. A 5′ RNA adapter (5′-GUUCAGAGUUCUACAGUCCGACGAUC-3′, Illumina) was ligated to the cleavage products, which contains a 5′ monophosphate. The ligated products were reverse-transcribed into cDNA using an oligo (dT) primer (5′-CGAGCACAGAATTAATACGACTTTTTTTTTTTTTTTTTT-3′, Operon) by SuperScript III RTase (Invitrogen), and amplified by PCR [(98°C for 30 s, 58°C for 30 s, and 72°C for 5 min, 8 cycles), 72°C for 7 min] with a pair of cDNA primers (forward, 5´-GTTCAGAGTTCTACAGTCCGA-3′ and reverse, 5´-CGAGCACAGAATTAATACGACT-3′, Operon) using Phusion polymerase (NEB). The resulting product was digested with restriction enzyme Mme I (NEB) to capture ~20 bp fragments from the 5´ end of double stranded cDNA. The digested products were ligated with an annealed duplex DNA adapter (top, 5´-p- TGGAATTCTCGGGTGCCAAGG- 3′ and bottom, 5´-CCTTGGCACCCGAGAATTCCANN-3′, Operon) using T4 DNA ligase (NEB). The ligated dsDNA products (~62 bp) were isolated using a 12% polyacrylamide (PAGE) and the purified products were amplified by PCR [98°C for 30 s, (98°C for 10 s, 58°C for 30 s, and 72°C for 20 s, 20 cycles), 72°C for 10 min) with a set of indexed TruSeq primers (forward, RP1 and reverse, RPI 1-4 for each library, Illumina). The final PCR products were purified by running 6% PAGE gel based on size (~128 bp). The quality and concentration of cDNA libraries were evaluated as described above. Libraries were used for throughput sequencing on an Illumina Miseq platform.

#### For degradome-seq analysis, the adaptor sequences, tRNA/rRNA sequences and low complexity sequences were removed from raw reads. The length of degradome sequences and small RNA sequences were trimmed to 20-21 nt and 19-24 nt, respectively. Any sequences without a match to the soybean genome (GLYMA1, Ensembl) were removed from further analysis. The potential targets of small RNAs were identified and validated using the UEA small RNA workbench and PAREsnip pipeline under a high stringency setting with the *Glycine max* reference genome (GLYMA1, Ensembl) and the known miRNAs of *Glycine max* (miRBase 21).

#### Transcriptome library construction, sequencing, data analysis

#### Total RNA was extracted using a mirVana miRNA isolation kit (Ambion). The quantity and integrity of total RNA were assessed using a RNA 6000 Nano kit on the Bioanalyzer 2100 system (Agilent). An input of 1 µg of total RNA was used to construct cDNA libraries using TruSeq Stranded Total RNA Sample Prep Kit (Illumina) with Ribo-Zero rRNA depletion kit following the manufacturer's instructions. The constructed libraries were analysed for size distribution and quality assessment on an Agilent 2100 Bioanalyzer (Agilent). The libraries were quantified essentially as described above.

#### For RNA-seq analysis, raw sequences were aligned to the soybean reference transcriptome (GLYMA1, Ensembl genes and transcripts) using the Strand NGS software (Strand Life Sciences, version 2.1) following the RNA alignment and RNA-seq analysis pipeline with standard parameters. Only reads with high quality scores were retained for further analysis.

#### RNA Blot Analysis

Small RNA fractions (20 µg) were separated on a denaturing 15% polyacrylamide (19:1 acrylamide/bisacrylamide) gel with 1 × TBE containing 8 M urea, and transferred to a positively charged nylon membrane (Roche) in 1 × TBE at 200 mA for 1 h. The RNA was cross-linked to the membrane by 1-ethyl-3-(3-dimethylaminopropyl) carbodiimide (EDC) as previously described [[47](#_ENREF_47)]. Pre-hybridization and hybridization were carried out in UltraHyb Oligo buffer (Ambion) at 50 °C for 1 hr and overnight, respectively. After hybridization, the membranes were washed twice in a low stringency buffer solution (2 × SSC, 0.1% SDS) at 50 °C for 5 minutes, then twice in a high stringency buffer solution (0.1 × SSC, 0.1% SDS) at 50 °C for 15 minutes. Probe detection was carried out using the DIG Detection Kit (Roche) according to the manufacturer's protocol. The membrane was scanned with a ChemiDoc XRS imaging system (Bio-Rad) or exposed to an X-ray film (Agfa curix). Soybean U6 small nuclear RNA (snRNA) was used as an internal control to normalize miRNA accumulation.

To detect mRNA cleavage products, a 247-bp fragment corresponding to the 3' region of Glyma05g01180.1 from the miR4376 cleavage site was amplified by RT-PCR with total RNA extracted from leaves of the soybean cultivar ‘Williams 82 (*rsv*)’ using specific primers (Table S1). The PCR product was cloned into pCR4Blunt-TOPO (Invitrogen) to generate construct pCR4-Glyma05g01180-mRNA. The insert was verified by sequencing. The constructed plasmid served as a template to generate RNA probes via *in vitro* transcription using a MAXIscript® T7/T3 Kit (Ambion) with DIG RNA labeling Mix (Roche) following the manufacturer’s instructions. For hybridization, total RNA was separated on a formaldehyde-permeated 1.2 % agarose gels and blotted to Hybond-N^+^ membranes (Amersham Biosciences). The membranes were pre-hybridized and hybridized with the UltraHyb ultrasensitive hybridization buffer (Ambion) according to the manufacturer’s instruction. The membranes were washed at 68 °C and the probes were detected as described above.

**RLM-5' RACE**

The poly (A)^+^ mRNAs were purified from total RNA using the NucleoTrap^@^ mRNA purification kits (Machery-Nagel) according to the manufacturer's protocol. Approximately 150 ng mRNAs were ligated to 5' RACE adaptor (5'-GCUGAUGGCGAUGAAUGAACACUGCGUUUGCUGG

CUUUGAUGAAA-3'). The ligated products were purified by spin column chromatography using mini quick spin RNA columns (Roche), and then reverse-transcribed into the first-strand cDNA using an antisense gene-specific (GSP) outer primer. PCR amplification was carried out using the 5' RACE outer primer (5'-GCTGATGGCGATGAATGAACACTG-3') and GSP outer primer. The initial PCR reaction was diluted (1/50) and used for nested PCR with the 5' RACE outer primer (5'-CGCGGATCCGAACACTGCGTTTGCTGGCTTTGATG-3') and GSP inner primer. The GSP outer and inner primers were shown in the supplementary table (Table S1). RACE fragments were cloned into pCR-BluntII-TOPO vector (Invitrogen) and sequenced.
